# A Cross-Sectional Study on Problematic Media Use (PMU) in Indian Children and Its Association With Behavioral Issues and Parenting Styles During the COVID-19 Pandemic Lockdown

**DOI:** 10.7759/cureus.79809

**Published:** 2025-02-28

**Authors:** Sreelakshmi Vaidyanathan, Suyog V Jaiswal

**Affiliations:** 1 Psychiatry, All India Institute of Medical Sciences, Nagpur, Nagpur, IND

**Keywords:** children and adolescents, covid-19, digital media use, parenting style, screen time

## Abstract

During the COVID-19 pandemic, social distancing and restrictions on the people's movement were imposed to curb the spread of COVID-19. With significant social restrictions, digital media emerged as the key to socialization and daily functioning. Psychological and behavioral issues have been reported in children with excessive screen use. A cross-sectional online study was conducted to evaluate problematic media use (PMU) among children, its impact on behavior, and its association with parenting styles.

Data were recorded using a questionnaire for sociodemographic, screen use details and behavioral issues (temper tantrums, disruptive behavior, sleep issues, etc.), Problematic Media Use Measure Short Form (PMUM - SF), and Parenting Styles & Dimensions Questionnaire - Short Version (PSDQ - SF).

A total of 253 parents responded, and 218 completed questionnaires were analyzed (mean age = 9.29; SD = 3.07). Sixteen percent (N=35) of the population showed PMU, most of whom (62.86%) belonged to the 6-to11-year age group. PMU was significantly associated with behavioral issues in the form of temper tantrums (p<0.01), clingy/attention-seeking behavior, sleep disturbances, and appetite issues (p<0.05). Authoritarian (p=0.001) and permissive (p=0.000) parenting styles were significantly higher in children with PMU. The study provides a snapshot of the situation during the initial phases of the pandemic regarding PMU in children and shows its behavioral impact. It also highlights the importance of parenting styles and the possibility of developing parental skills to curtail PMU.

## Introduction

The lockdown during the COVID-19 pandemic, implemented in India in March 2020, brought unprecedented changes to daily life. With educational institutions closed and children confined to their homes, digital media became an essential tool for education, communication, and entertainment. This surge in screen time has raised significant concerns about problematic media use (PMU) among children and adolescents, with studies highlighting its adverse effects on mental, emotional, and behavioral health [1,2].

PMU is defined as an excessive and compulsive use of digital media that interferes with daily activities, social relationships, and psychological well-being [3]. PMU encompasses behaviors such as prolonged screen time, withdrawal symptoms when not using media, and the inability to control media consumption [4]. During the COVID-19 lockdown, PMU in children escalated due to restricted outdoor activities, leading to concerns about addiction-like behaviors, sleep disturbances, and emotional dysregulation [5].

Recent research emphasizes the negative consequences of excessive media use, including sleep disturbances, heightened anxiety, attention deficits, and increased aggression [6,7]. Studies in the Indian context have reported an alarming rise in digital addiction during the lockdown, with direct implications in children’s psychological well-being and academic performance [8]. This calls for a deeper examination of factors contributing to PMU, particularly the role of parenting styles.

Parenting approaches significantly influence children’s screen time and digital habits. Authoritative parenting, characterized by warmth and structure, has been associated with lower PMU levels, while permissive parenting often correlates with higher screen dependency [9]. High-quality parent-child interaction and warmth in their relationship have been associated with lower digital media use and addiction [10]. Given that Indian families spent more time together during the lockdown, this period presents a unique opportunity to study the intersection of parenting styles, children’s media consumption, and related behavioral issues.

However, existing literature predominantly focuses on global contexts, with limited studies exploring this dynamic in India. Research by Sharma et al. [11] and Sharma et al. [12] highlights this gap, emphasizing the need for culturally relevant investigations into PMU and its psychosocial impacts on Indian families.

This study was conducted during the first COVID lockdown and aimed to (i) evaluate the prevalence of PMU among school-going children and adolescents in India during the COVID-19 lockdown, (ii) examine its association with behavioral issues such as temper tantrums, sleep disturbances, etc. and (iii) explore the influence of parenting styles on PMU.

By addressing these dimensions, this research contributes to the growing body of literature on digital media use among children and offers insights into parenting strategies that can mitigate the risks associated with PMU.

## Materials and methods

This study was approved by the Institutional Ethics Committee of AIIMS Nagpur. It was an observational cross-sectional study conducted in May 2020, during the COVID-19 pandemic. Given the social restrictions in place at the time, an online data collection methodology was adopted. Data were collected through a Google Form designed for this purpose, which was active for a time-bound period of one month. No formal sample size calculation was employed due to the time-sensitive nature of the study, aimed at capturing the immediate impact of lockdown measures on children's screen time during the pandemic. All responses submitted within this month were accepted.

Parents of school-going children, including those attending playschools, who understood the contents of the questionnaire, had basic proficiency in using online forms, and were willing to participate, were included in the study. Children with a history of neurodevelopmental or neurological disorders, such as attention deficit hyperactivity disorder (ADHD), autism spectrum disorder, intellectual disability, or cerebral palsy, as reported by the parent, were excluded, except for those with specific learning disabilities.

Data collection utilized a snowball sampling technique. Initial participants were recruited through social media outreach and parenting forums the investigators were a part of, ensuring a diverse pool. Snowballing was conducted by requesting participants to share the survey with others in their network. The study did not impose strict geographic limits, though responses were predominantly urban, likely due to better internet accessibility. No incentives were offered for participation. Participants were also given the option to seek assistance and were informed of the availability of the study results. The Google Form had settings that limited responses to one per participant and remained active for a month. The form included a landing page for parental/caretaker consent, followed by the questionnaire.

The sections in Google Forms were as follows: (i) Information regarding the study followed by explicit consent document; the rest of the sections were presented only when the person consented to participate in the study; (ii) Semi-structured questionnaire for demographic details of age, gender, class of child, type of family, behavioral issues, details of screen use - parents reported their child’s daily screen time (in hours) across various devices (TV, smartphone, tablet, etc.) - and parental concern for the same; (iii) Problematic Media Use Measure Short Form (PMUM-SF): This is a nine-item parent report measure of screen media addiction [13]. Screen media was defined as “any type of media that the child uses that has a screen, such as television, video games, tablets, laptops, computers, smartphones, handheld video games, etc.”. Responses are on a 5-point Likert scale ranging from Never (1) to Always (5). The scale was developed from items generated based on DSM-5 criteria for Internet Gaming Disorder and has been shown to have a high internal consistency (Cronbach’s α = 0.93). A score of ≥3 for 5 out of 9 questions was considered PMU based on high reliability, alignment with DSM-5 criteria, and available Indian literature supporting its use in distinguishing problematic from non-problematic media use [14,15]; (iv) Parenting Styles & Dimensions Questionnaire - Short Version (PSDQ - SF): This is a 32-item parent self-report based on Baumrind’s typology (Authoritative, Authoritarian and Permissive parenting styles) [16]. It is a modified version of the original 62-item PSDQ and has been widely used in studies on parents of school-going children. It is designed as a parental self-report and is measured on a 5-point Likert scale ranging from Never (1) to Always (5). Adequate internal consistency and reliability have been demonstrated, with Cronbach’s α reported to be 0.86, 0.82, and 0.64 for Authoritative, Authoritarian, and Permissive Parenting scales, respectively [16].

Both these scales have been used previously in Indian population samples [14,17].

The responses were documented online and automatically stored in a secure Google Drive spreadsheet, accessible only to the study investigators. Data collected through Google Forms over one month were exported to Microsoft Excel 2019 (Microsoft Corporation, Redmond, USA) for data cleaning and coding and subsequently imported into IBM SPSS Statistics for Windows, Version 20 (Released 2011; IBM Corp., Armonk, New York, United States) for analysis.

**Figure 1:**
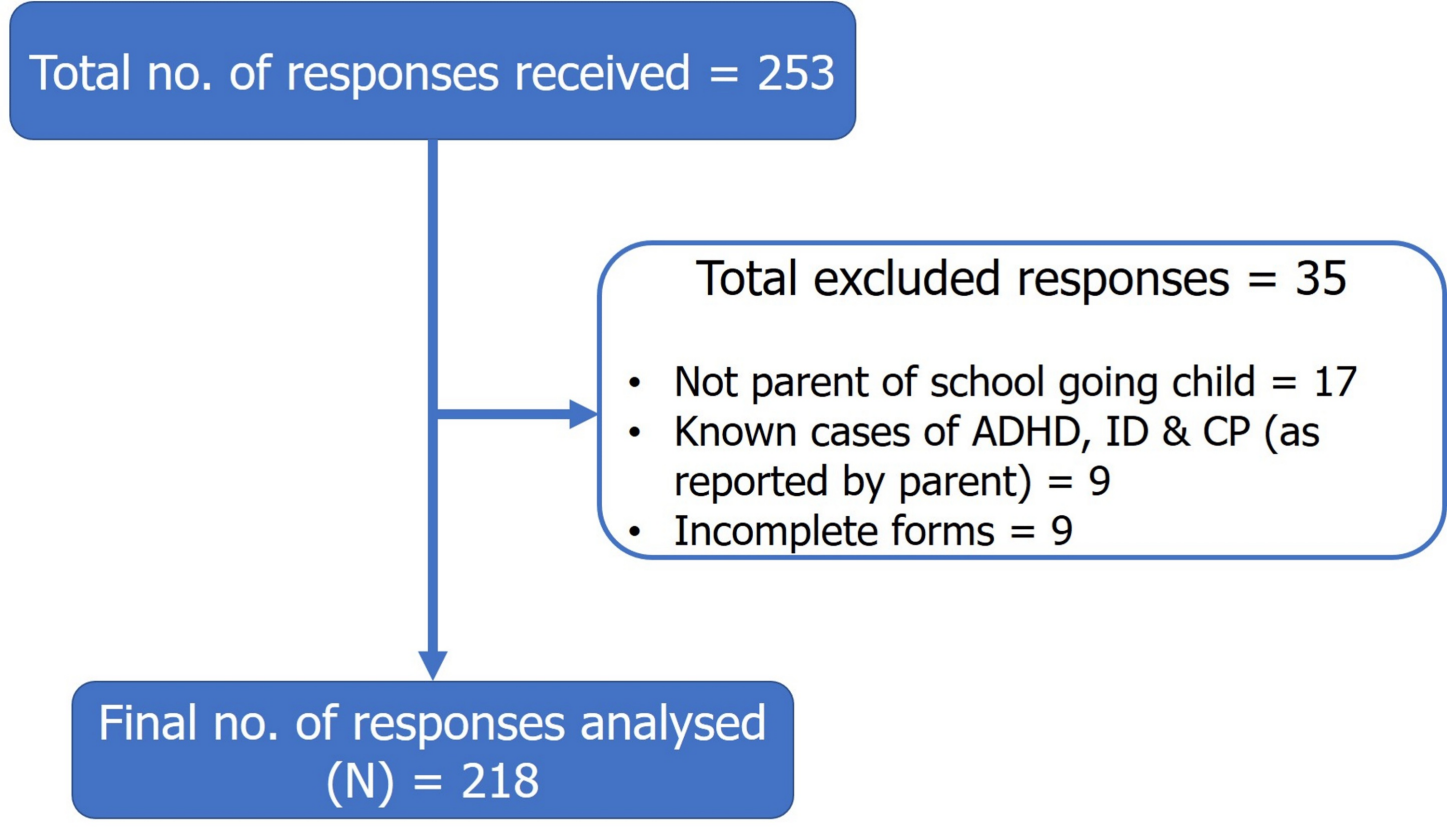
Flowchart showing data collected and analyzed ADHD: Attention Deficit Hyperactivity Disorder; ID: Intellectual Disability; CP: Cerebral Palsy

A total of 253 responses were received, of which 35 were excluded with 218 responses being included in the final analysis (Figure 1). These exclusions were made due to incomplete forms (N=20), parent-reported neurodevelopmental conditions (N=10), and responses from parents of non-school-going children (N=5). This exclusion strategy helped ensure data integrity and relevance.

Descriptive statistics, including percentages, were calculated for sociodemographic variables, parent reported behavioral issues and screen use details, and scores from the PMUM-SF and PSDQ-SF scales. Chi-square tests and t-tests were performed to examine associations between PMU, behavioral issues, and parenting styles. Statistical significance was set at p < 0.05 (two-tailed).

## Results

Most responses (72%) were completed by the mothers. The mean age of children was 9.29 years (S.D. = 3.07), and the majority were aged between 6 and 11 years (middle childhood) (Table 1). The male and female children who experienced almost PMU were present in 16% of the sample (n=35), with the majority (62.86%, n-22) in the 6-to-11-year-age group, followed by 12-to-18-year age group (31.42%, n-11) and 2-to-5 years (5.71%, n-2). No significant association of PMU was found with the family type, age, gender, or class of the child. PMU was significantly associated with behavioral issues with more temper tantrums, clingy/attention-seeking behavior, sleep, and appetite issues in children with PMU than in those without (Table 2). Authoritarian and permissive parenting styles were significantly associated with PMU, whereas authoritative parenting showed no such association (Table 3).

**Table 1:** Demographic details of the sample (N: 218)

		N
Parent	Father	61 (28%)
	Mother	157 (72%)
Family type	Nuclear	138 (63.3%)
	Joint	80 (36.7%)
Child’s gender	Male	110 (50.5%)
	Female	108 (49.5%)
Age of child	(2-5 years) Early childhood	11 (5%)
	Middle childhood (6-11 years)	152 (69.7%)
	Early adolescence (12-18 years)	55 (25.2%)
School standard	Preschool	09 (4.1%)
	Primary school	110 (50.5%)
	Middle school	60 (27.5%)
	High school	39 (17.9%)

**Table 2:** Association of problematic media use (PMU) with behavioral issues * Significance at <0.05 level; **Significance at <0.01 level

Variable		PMU N=35	No PMU N=183	p-value	phi
Temper tantrums	Yes	29	57	.000**	-.388
	No	6	126		
Disruptive behavior	Yes	7	19	.186	-.109
	No	28	164		
Clingy/attention seeking behavior	Yes	18	59	.047*	-.147
	No	17	124		
Sleep issues	Yes	23	79	.024*	-.166
	No	12	104		
Change in food habits	Yes	12	40	.173	-.107
	No	23	143		
Appetite issues	Yes	15	42	.025*	-.166
	No	20	141		

**Table 3:** Association of problematic media use (PMU) with parenting style **Correlation is significant at 0.01 level PSDQ: Parenting Styles & Dimensions Questionnaire

PSDQ scale	PMU	PSDQ scale mean score (S.D.)	T	p	Mean difference	95% CI
Authoritative style	Yes	3.98 (.64)	.975	.331	.112	-.11 to .34
	No	4.09 (.62)				
Authoritarian style	Yes	2.54 (.55)	3.49	.001**	-.412	-.64 to -.18
	No	2.12 (.65)				
Permissive style	Yes	2.94 (.80)	4.69	.000**	-.587	-.83 to -.34
	No	2.35 (.65)				

## Discussion

This study provides a snapshot of media use among school-going children one month into the COVID-19 lockdown. The sudden shift to digital dependence significantly altered children's routines, reinforcing long-term implications for behavioral health and family dynamics. Our findings align with recent global studies, emphasizing the complex interplay between PMU, behavioral dysregulation, and parenting styles. 

PMU was present in 16% of the sample (n=35), with the majority (62.86%) in the 6-to-11-year age group, followed by the 12-to-18-year group, and the 2-to-5-year group. Despite previous studies reporting age and gender-based differences in PMU prevalence, our study found no significant association between PMU and family type, age, gender, or class. This aligns with findings by Rodríguez-Rojo et al. who reported that PMU risks are shaped more by environmental and behavioral factors than demographic variables [17].

The primary screen medium used was television, with excessive screen time most common in children aged 6-11 years. Our findings are consistent with those by Pasi et al. [1] and Madigan et al. [18], who noted that early-school-age children had the highest screen engagement, especially before structured online learning was widely implemented. Though pre-pandemic screen habits and parental screen use were factors beyond the scope of this study and the screen use details were as reported by parents, this level of screen use goes way beyond the recommended usage for this age group as per the available guidelines [19,20].

Our findings confirm a significant association between PMU and behavioral problems, with higher rates of temper tantrums, clingy/attention-seeking behavior, sleep disturbances, and appetite issues among children with PMU (Table 2). These results align with findings in previous literature [10,21-24]. Coyne et al. found that PMU disrupts emotional regulation, leading to increased temper tantrums and attachment-seeking behaviors [25]. On the other hand, high impulsivity and difficulty in emotion regulation have also been shown to be associated with PMU [26,27]. Children with deficits in behavioral response regulation may be more prone to developing problematic use because of the difficulty in balancing the rewarding aspects of media use with other activities or goals [10]. Zhang et al. reported that parental technoference, which is frequent parental engagement in digital activities, contributed to disrupted sleep patterns in children with PMU [28]. Li et al. found that maternal stress and permissive screen-time parenting were linked to appetite changes in children with excessive screen exposure [29]. While previous research suggested a mixed relationship between PMU and appetite issues, our findings indicate that children with PMU exhibited more appetite disturbances. One possible explanation is that increased digital engagement interfered with natural hunger cues and structured mealtime routines, a phenomenon previously described by Eales et al. in a longitudinal study on screen use habits [30]. It is important to note that, during the pandemic, there was a transient change in the dietary habits of the people owing to multiple factors from reduced market timings to the availability of fresh foods. The stress-related eating and unconscious snacking during screen use may be an important mediator in the relationship between appetite issues and PMU.

Parenting styles play a significant role in shaping children's digital behaviors. Our study confirms that authoritarian and permissive parenting styles were significantly associated with PMU, whereas authoritative parenting showed no such association. These patterns are well-supported by recent research. In the context of authoritarian parenting and PMU, the strict control in parenting style was reported to increase covert screen use by children as a rebellion against rigid restrictions [29] while the resultant higher emotional distress prompted children to use digital media as a coping mechanism [31]. Permissive parenting with low regulation on the other hand was found to encourage excessive, unregulated screen time, leading to habitual overuse [32]. Oliveira et. al. reported these parents tend to use digital media as a pacifier, thereby reinforcing dependency [33]. Authoritative parenting unlike the above two parenting styles promotes self-regulation and consistent digital habits and provides structured, balanced screen use policies reducing the likelihood of problematic engagement and may explain why no significant association with PMU was observed in our study [34].

Additionally, Ereskici et al. found that parenting awareness of digital risks significantly reduced PMU prevalence, reinforcing the need for structured screen-time education programs for parents [32]. Baminiwatta et. al. reported maternal self-efficacy in managing screen use among children to be associated with lower PMU [35]. A consensus on the prevention of problematic Internet use during the COVID-19 pandemic also emphasized the parental role in information and communications technology (ICT)-related behaviors of their children and encouraged active involvement in the regulation of child usage and their own use (as role models) [36].

While this study provides valuable insights into PMU and its behavioral associations, potential confounders and limitations need to be acknowledged. The study was done in a unique time of pandemic and the impact of the PMU on the long run requires detailed deliberation. The cross-sectional study design limits the ability to infer causal relationships between PMU and behavioral issues. It remains unclear whether excessive screen use leads to behavioral problems or whether children with pre-existing behavioral difficulties are more likely to engage in PMU. Second, as the study only looked at the time period immediately after the lockdown was instituted, further clarity is required as to whether these trends are transitory or persistent. Third, the study relies on parent-reported data, which may introduce recall bias and social desirability bias, as parents might overestimate or underestimate their children’s screen time and behaviors. Lastly, while parenting style was examined as a key factor, other potential cultural and environmental factors, such as socioeconomic status, family digital literacy and household digital access, parental screen habits, parental stress levels, family environment, school engagement, and availability of alternative activities, may have influenced PMU patterns but were not fully explored.

It will be imperative to study further the causal relationship between screen use and behavioral issues in children. The comparison of screen use between children with and without behavioral issues may be a good start to deciphering the causality. Furthermore, an interventional study in children with behavioral issues which studies the problematic media use before and after the intervention gives clue about the causality. The study of screen use with objective measures such as monitoring of the use of screen, type of applications, and times of use in smart devices will help in getting the data more accurately than the subjective report as done in the current study. Parental interventions establish structured screen-time rules at home, modeling healthy digital habits, and integrating media literacy education into parenting programs.

Addressing these limitations in future studies, by including objective screen time tracking, diverse socioeconomic samples, and longitudinal data collection, would enhance the robustness of findings and provide more definitive insights into the long-term impact of PMU. Interventional studies that include coaching on parenting skills and emotional regulation in children may provide further insight and pave the way to policies that can be adapted to cut down on PMU at large. The digital wellness programs at the school level as well as national guidelines for screen use for parents as well as children will be helpful in addressing the issue of PMU. 

Recommendation for future research and interventions

To mitigate PMU risks, future research and interventions should focus on: (i) Longitudinal studies tracking the sustained impact of PMU on child development; (ii) Parenting-focused interventions promoting digital literacy, modelling healthy digital habits, integrating media literacy into parenting programs and structured media supervision; (iii) Holistic strategies that integrate behavioral therapy, digital parenting guidance, school-based digital wellness programs. and child mental health support.

## Conclusions

The COVID-19 pandemic catalyzed a significant shift in children's media use, with long-term behavioral and psychological consequences. During the lockdown, many children exhibited PMU, with higher rates of behavioral issues such as temper tantrums, attention-seeking behaviors, sleep disturbances, and appetite problems. Parenting styles significantly influenced PMU, with authoritarian and permissive parenting linked to higher PMU, while authoritative parenting showed no association.

As digital media becomes an integral part of modern childhood, balanced parental mediation and structured screen-use policies are essential for fostering healthy media habits while minimizing PMU-related risks.

## Appendices

Questionnaire

Google form link: https://forms.gle/ajMWvb1YoxTuM2fJ7
